# *Desulfotomaculum varum* sp. nov., a moderately thermophilic sulfate-reducing bacterium isolated from a microbial mat colonizing a Great Artesian Basin bore well runoff channel

**DOI:** 10.1007/s13205-011-0017-5

**Published:** 2011-08-09

**Authors:** Christopher D. Ogg, Bharat K. C. Patel

**Affiliations:** Microbial Gene Research and Resources Facility, School of Biomolecular and Physical Sciences, Griffith University, Brisbane, QLD 4111 Australia

**Keywords:** *Desulfotomaculum varum* sp. nov, Phylum *Firmicutes*, Great Artesian Basin, Australia, Phylogeny, Taxonomy

## Abstract

A strictly anaerobic moderately thermophilic bacterium, designated strain RH04-3^T^ (T = type strain), was isolated from a red colored microbial mat that colonizes a Great Artesian Basin (GAB) bore well (Registered Number 17263) runoff channel at 66 °C. The cells of strain RH04-3^T^ were straight to slightly curved, sporulating, Gram-positive rods (2.0–5.0 × 1.0 μm) that grew optimally at 50 °C (temperature growth range between 37 and 55 °C) and at pH 7 (pH growth range of 5.0 and 8.5). Growth was inhibited by NaCl concentrations ≥1.5% (w/v), and by chloramphenicol, streptomycin, tetracycline, penicillin and ampicillin. The strain utilized fructose, mannose, glycerol, lactate, pyruvate and H_2_ in the presence of sulfate, and fermented pyruvate in the absence of sulfate. Strain RH04-3^T^ reduced sulfate, sulfite, thiosulfate and elemental sulfur, but not nitrate, nitrite, iron(III), arsenate(V), vanadium(V) or cobalt(III) as terminal electron acceptors. The G + C content of DNA was 52.4 ± 0.8 mol % as determined by the thermal denaturation (Tm) method. 16S rRNA sequence analysis indicated that strain RH04-3^T^ was a member of the genus *Desulfotomaculum* and was most closely related to *Desulfotomaculum putei* (similarity value of 95.2%) and *Desulfotomaculum hydrothermale* (similarity value of 93.6%). On the basis of phylogenetic and phenotypic characteristics, strain RH04-3^T^ is considered to represent a novel species of the genus *Desulfotomaculum*, for which the name *Desulfotomaculum varum* sp. nov. is proposed. The type strain RH04-3^T^ = JCM 16158^T^ = KCTC 5794^T^.

## Introduction

The Great Artesian Basin (GAB) underlies arid and sub-arid regions of approximately 22% of the Australian continent and is considered to be the world’s most expansive geothermal aquifer (Habermehl 1980). The GAB has an estimated water-storage capacity of 8.7 × 10^12^ m^3^ and, since 1878, more than 5,000 bore wells have been drilled into the GAB to provide a vital water resource for outback Australian communities. Several gas and oil fields are located in the GAB aquifer, and the Queensland State Government and the Australian Commonwealth Government have approved the exploitation of these resources. The GAB aquifer therefore not only supports the domestic activities of over 180,000 outback rural residents, but also supports agriculture-, mineral-, gas- and petroleum-production industries that generate a combined income of approximately $4 billion (AUD) per annum to the Australian economy. A significant decrease in GAB bore well pressure and outflow volume has been observed in recent years; since 1999, the Australian Commonwealth Government has invested heavily in a program to cap and control bore wells in order to restore the pressure of the basin. Despite these efforts, water loss from leaky corroded bore wells still occurs, and if this remains unchecked may cause detrimental long-term effects to the future of water storage, pressure and economic value of the GAB.

It is well documented that sulfate-reducing bacteria (SRB) exert an immense influence on the geochemical properties of deep subsurface environments. Thermal environments including subsurface environments that are heated due to their depth are known to harbor a diverse range of thermophilic SRB belonging to the genera *Desulfotomaculum* (Campbell and Postgate 1965), *Thermodesulfotobacterium* (Zeikus et al. 1983), *Thermodesulfovibrio* (Henry et al. 1994), *Thermodesulforhabdus* (Beeder et al. 1995), *Thermodesulfobium* (Mori et al. 2003), *Thermodesulfatator* (Moussard et al. 2004), *Desulfothermus* (Kuever et al. 2005), *Desulfovirgula* (Kaksonen et al. 2007a), *Desulfurispora* (Kaksonen et al. 2007b) and *Thermosulfidibacter* (Nunoura et al. 2008). We have previously speculated that the co-colonization of indigenous microbial communities of SRB on the metal-casing of bore wells could significantly increase corrosion processes, and this may eventually lead to total bore well failure. We have previously reported on *Desulfotomaculum australicum,* a thermophilic SRB isolated from the GAB (Love et al. 1993). In this report, we extend our studies of novel SRB from the GAB and describe a strictly anaerobic, moderately thermophilic, SRB, designated strain RH04-3^T^, which represents a new species of the genus *Desulfotomaculum*, for which the name *Desulfotomaculum varum* is proposed.

## Materials and methods

### Sample source and strains

A 66 °C red-colored microbial mat sample was collected from the run-off channel of the New Lorne Bore well (RN 17263) located near the township of Blackall, some 1,000 km northwest of Brisbane, Queensland State, Australia (24°54′48′′S and 145°08′18′′E), and was used in previous studies (Ogg and Patel 2009; Spanevello 2001).

### Enrichment and isolation

Enrichment and isolation were performed in a modified anaerobic Patel Laboratory (PL) medium, which contained (l^−1^ deionized water): 1 g NH_4_Cl, 0.6 g K_2_HPO_4_, 0.3 g KH_2_PO_4_, 0.1 g MgCl_2_.6H_2_O, 1.0 g NaCl, 12.0 g HEPES and 0.2 g yeast extract. The medium was adjusted to pH 7.2, heated in an autoclave at 121 °C and 1–1.5 kg cm^−2^ pressure for 10 min to remove dissolved oxygen, cooled under a stream of oxygen-free nitrogen gas, and 1 ml vitamin solution l^−1^ (Wolin et al. 1963), 1 ml trace-element solution l^−1^ (Zeikus et al. 1979) and, unless indicated otherwise, 2 ml of 10% Na_2_S were added. The medium was then dispensed into Hungate tubes under oxygen-free nitrogen gas (Patel et al. 1985) and sterilized for 20 min at 121 °C and 1–1.5 kg cm^−2^ pressure. After sterilization, sulfate was added from a sterile anaerobic 1 M stock solution to a final concentration of 20 mM. When sulfate was added, the medium was designated SO_4_-PL medium.

For enrichment studies, 20 μl of sterile anaerobic stock solutions of electron donors including extracts, carbohydrates, organic acids and alcohols (10%) and aromatic compounds (200 mM) listed in Table 1 were dispensed into wells of U-bottom deep-well (1.2 ml) microtiter plates (Sarstedt, Germany). The solutions were dried at 50 °C for 1 h and stored inside an anaerobic chamber (Coy Laboratory Products, USA) for at least 24 h to remove any trapped oxygen prior to use. A 5% suspension of the red mat sample was prepared in SO_4_-PL medium lacking Na_2_S and 1 ml inoculated into each well under anaerobic conditions in the anaerobic chamber. The plates were then placed horizontally inside an anaerobic gas jar (Oxoid Ltd., UK), the jars removed from the anaerobic chamber and incubated at 50 °C. After 3 days of incubation, the plates were returned to the anaerobic chamber, cooled for 1 h, and 50 μl of 10% freshly prepared sterile anaerobic stock solution of Fe(NH_4_)_2_(SO_4_)_2_ added to each well to give a final concentration of 0.5%. Wells in which black precipitates of ferrous sulfide were produced were suggestive of sulfate reduction and scored as positive for metabolism. Cultures from the positive wells were serially diluted in Hungate tubes containing SO_4_-PL medium and the respective enrichment carbon source and incubated at 50 °C for 3 days, after which the tube containing the highest dilution was selected and the procedure of serial dilution repeated at least a further two times. A shake tube method was used to isolate pure cultures. For this, the culture was serially diluted in Hungate tubes containing the respective energy substrate amended SO_4_-PL medium, fortified with 1% gelrite (as a solidifying agent), and well-isolated colonies that developed after 7 days incubation at 50 °C were picked and subcultured into fresh SO_4_-PL medium amended with the appropriate carbon source. Pure cultures were preserved in a 50:50 mixture of glycerol: SO_4_-PL medium amended with the appropriate carbon source and stored at −20 °C.

**Table 1:** Results of sulfate reduction with different electron acceptors in enrichment cultures

Growth substrate (concentration)	Sulfate reduction
Extracts (0.2%):	
Tryptone	++
Peptone	++
Alcohols (0.2%):	
Ethanol	**–**
Glycerol	++
Organic acids (0.2%):	
Acetate	**–**
Propionate	**–**
Formate	+
Pyruvate	++
Malonate (5 mM)	**–**
Adipate (5 mM)	**+**
Aromatic compounds (4 mM):	
Benzoate	**–**
3-Hydroxybenzoate	**–**
4-Hydroxybenzoate	**–**
2,5-Dihydroxybenzoate	**–**
3-Chlorobenzoate (2 mM)	**–**
Phenylacetate	**–**
4-Hydroxyphenylacetate	**–**
Cinnamate	**–**
4-Hydroxycinnamate	**–**
2-Hydroxycinnamate	**–**
2-Methoxybenzoate	+^a^
4-Methoxybenzoate	+^a^
Vanilline	**–**
Syringate	**–**
Control (no substrate)	**–**

A deep-well U-bottom microtiter plate was prepared with various substrates listed below and sulfate reduction determined using the methods described in the text

Sulfate reduction is scored as: ++ strong, + weak, − no sulfate reduction

^a^Subsequent subculture of the enrichment failed to grow

### Microscopy

Cell morphology of strain RH04-3^T^ was determined by phase-contrast microscopy and electron microscopy (Kanso and Patel 2003).

### Optimum growth conditions

The growth of strain RH04-3^T^ was tested at temperatures ranging from 26 to 65 °C and pH values ranging from 4.0 to 9.5.

### Studies on electron donor and acceptor

The ability of strain RH04-3^T^ to utilize fructose, mannose, glycerol, lactate, pyruvate, glucose, lactose, ethanol, malate, acetate, propionate, formate, succinate, casamino acids, benzoate, butyrate, propanol, methanol and butanol was tested in PL medium with or without 20 mM sodium sulfate. For this, the substrates from sterile anaerobic stock solutions (10%) were added to the medium at a final concentration of 0.2%. The ability of strain RH04-3^T^ to use H_2_ or CO_2_ was tested in 30-ml serum bottles containing SO_4_-PL medium with or without 2 mM acetate, under H_2_ (100%) or CO_2_ (100%) gas phase. SO_4_-PL medium lacking the respective substrate was used as a negative control. Substrate utilization was determined as positive when growth and/or sulfate reduction was detected.

Sodium sulfate (20 mM), sodium thiosulfate (20 mM), sodium sulfite (5 mM), elemental sulfur (1%), sodium nitrate (20 mM), sodium nitrite (5 mM), fumarate (20 mM), ammonium iron(III) citrate (0.2%), sodium arsenate (5 mM), sodium metavanadate (4 mM) and sodium cobalt(III) (5 mM) were tested as electron acceptors in PL medium amended with 0.2% glycerol. Na_2_S was excluded from the medium when testing for iron(III), vanadium(V), arsenate(V) and cobalt(III) reduction.

### Analytical studies

Unless indicated otherwise, all experiments were conducted at least twice in triplicate, and cultures were subcultured at least once in the same medium prior to the conduct of the experiment. All growth studies were performed in SO_4_-PL medium amended with 0.2% glycerol and incubated for up to 14 days unless otherwise indicated. Growth was measured at 600 nm by inserting Hungate tubes directly into a modified cuvette holder of a Novaspec LKB spectrophotometer (Pharmacia-Biotech Pty. Ltd). Electron acceptor utilization was determined by measuring growth and using colorimetric assays as described by Ramamoorthy et al. (2006), Sorensen (1982) and Ogg and Patel (2009). In addition, vanadium(V) reduction was inferred when a green media color and/or a green precipitate was observed, and confirmed using the vanadate assay as described by Carpentier et al. (2003). For this, 250 μl of 1% (wt/vol) diphenylcarbazide (DPC) in acetone was added to 250 μl of 2 M H_2_SO_4_, which was combined with 500 μl of diluted sample and the absorbance was measured at 320 nm after 15 min.

### Antimicrobial agents

Antibiotic sensitivity of strain RH04-3^T^ was determined by adding antibiotics from filter-sterilized stock solutions to a final concentration of 10 and 100 μg ml^−1^. To test for growth on NaCl (range between 0 and 10%), NaCl was weighed directly into Hungate tubes, with the medium dispensed and sterilized before use.

### Fatty acid composition

The whole-cell fatty acid composition of strain RH04-3^T^ was determined by DSMZ, using cells grown with lactate and sulfate as the electron donor and acceptor, respectively. In previous studies, the closest relatives of strain RH04-3^T^, *D. hydrothermale* DSM 18033^T^ and *D. putei* DSM 12395^T^, had been grown under the same conditions. Fatty acid methyl esters were prepared from 40 mg of cells using minor modifications of the method of Miller (1982) and Kuykendall et al. (1988), and were separated and analyzed using Sherlock Microbial Identification System (MIDI 1999).

### DNA isolation

High molecular weight (HMW) DNA was isolated using the previously described method (Marmur 1961; Ogg and Patel 2009).

### Determination of G + C content

High molecular weight (HMW) DNA of strain RH04-3^T^ was amplified as described, by using a TempliPhi Amplification Kit (Amersham Biosciences, UK) and the mol % G + C content was determined by the thermal denaturation method (Marmur and Doty 1962) in a Cintra20 spectrophotometer (GBC Scientific Equipment, Australia) as described by Ogg and Patel (2009).

### 16S rRNA sequence studies

16S rRNA gene amplification, sequencing and analysis were determined using the method of Ogg and Patel (2009). The GenBank Accession number for strain RH04-3^T^ = KCTC 5794^T^ = JCM 16158^T^ (T = type strain) is GU126374.

## Results

### Enrichment and isolation

Of the 24 wells that contained the electron donors peptone, tryptone, glycerol, formate, pyruvate, 2-methoxybenzoate, 4-methoxybenzoate and adipate (Table 1), 8 produced black precipitates of FeS following the addition of Fe(NH_4_)_2_(SO_4_)_2_, which could suggest that sulfate reduction had occurred. Subsequently, all enrichments with the exception of 2-methoxybenzoate and 4-methoxybenzoate were successfully subcultured and showed growth as well as sulfate reduction. We report in this paper, our studies on strains isolated from the tryptone and glycerol enrichments. 16S rRNA gene sequence analysis of 1,000 nucleotides of strain RG04, an isolate from the tryptone enrichment, revealed a 100% similarity to *Caloramator australicus* strain RC3^T^. We had previously reported on the isolation and characterization of *Caloramator australicus* strain RC3^T^ from the red mat habitat (Ogg and Patel 2009). Neither strain RG04 nor *Caloramator australicus* strain RC3^T^ reduce sulfate to sulfide, but rather produce hydrogen sulfide from tryptone metabolism, which reacts with Fe(NH_4_)_2_(SO_4_)_2_ to produce the black precipitate. Other thermophilic anaerobes such as *Thermoanaerobacterthermohydrosulfuricus* (formerly *Clostridiumthermohydrosulfuricum*) also produce hydrogen sulfide from tryptone, peptone and yeast extract metabolism and not from sulfate reduction (Lee et al. 1993). Given the extremely close phylogenetic similarity of strain RG04 to *Caloramator australicus* strain RC3^T^, further studies were not performed. Five strains, designated RH04-1, RH04-2, RH04-3, RH04-7 and RH04-8, were isolated from the glycerol enrichment culture. 16S rRNA sequence comparisons revealed that all five isolates had a similarity value of 100%. Furthermore, all strains were morphologically identical and had the same substrate utilization pattern and hence only one representative strain, RH04-3^T^, was designated the type strain and characterized further.

### Morphology

**Fig. 1:**
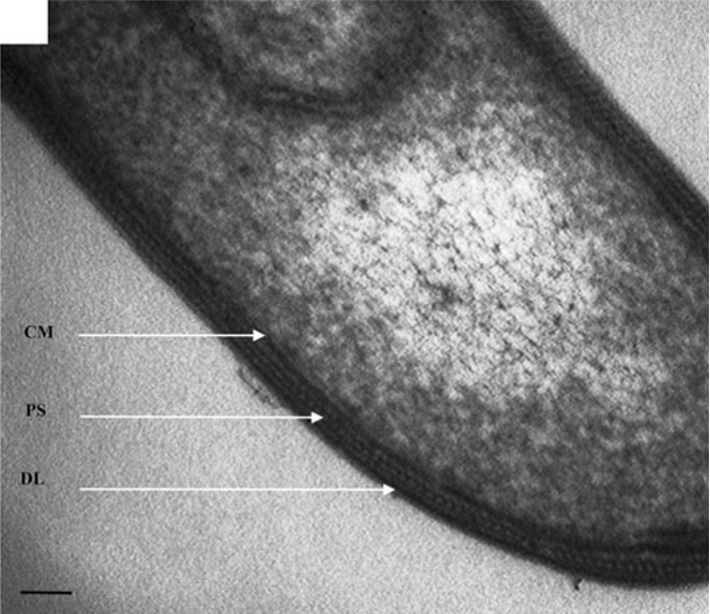
Transmission electron micrographs of a thin section of cells of strain RH04-3^T^ showing electron-dense protein subunits (PS) situated between an electron-dense cell membrane (CM) and an outer diffused layer (DL), typical of a Gram-positive cell wall ultrastructure. *Bar* 50 nm

The cells of strain RH04-3^T^ were straight to slightly curved rods (2.0−5.0 × 1.0 μm), which existed singly, in pairs and short chains, were peritrichously flagellated and regularly formed terminal to sub-terminal oval-shaped spores. Strain RH04-3^T^ stained Gram positive and electron micrographs of thin sections revealed cell walls composed of electron-dense protein subunits sandwiched between an electron-dense inner cell membrane and an outer diffused layer, typical of Gram-positive cell wall ultrastructure (Fig. 1) similar to *Desulfotomaculumaustralicum* (Love et al. 1993) and *Thermoanaerobacter brockii* (formerly *Thermoanaerobium brockii*) (Zeikus et al. 1979; Patel et al. 1986).

### Optimum growth conditions

Strain RH04-3^T^ grew optimally at 50 °C (temperature growth range between 37 and 55 °C) and a pH of 7 (pH growth range of 5.0 and 8.5) with 0.2% glycerol as the electron donor and 20 mM sulfate as the electron acceptor, and in sulfate-free PL medium amended with 0.25% pyruvate. Growth occurred in the absence of vitamins and trace elements, but required trace amounts of yeast extract for growth on carbon sources. Higher concentrations of yeast extract did not support growth in the absence of any other added carbon sources. Strain RH04-3^T^ did not grow under aerobic conditions in medium D (Brock and Freeze 1969), or in aerobically prepared PL medium amended with 0.2% pyruvate.

### Substrates and electron acceptor studies

Strain RH04-3^T^ utilized fructose, mannose, glycerol, lactate and pyruvate, but not glucose, lactose, ethanol, malate, acetate, propionate, formate, succinate, casamino acids, benzoate, butyrate, propanol, methanol and butanol. In the absence of sulfate, pyruvate was fermented, but not fructose, mannose, glycerol, lactate, acetate, malate, formate, ethanol, butanol or propanol. Strain RH04-3^T^ utilized H_2_ but not CO_2_, in the presence or absence of 2 mM acetate and sulfate.

Strain RH04-3^T^ reduced sulfate, sulfite, thiosulfate and elemental sulfur, but not fumarate, nitrate, nitrite, iron(III), arsenate(V), vanadium(V) or cobalt(III).

### Antimicrobial agents

Strain RH04-3^T^ was completely inhibited in the presence of 10 μg ml^−1^ chloramphenicol, streptomycin, tetracycline, penicillin and ampicillin. Strain RH04-3^T^ grew in the absence of NaCl, but not with NaCl concentrations greater than 1.5% (w/v) [NaCl growth range was between 0 and 10% (w/v)]. Increased growth was observed with 0.1% NaCl (w/v). Under optimum pH and temperature, strain RH04-3^T^ required at least 0.01% (w/v) yeast extract for growth.

### G + C content

The mol% G + C content of the genomic DNA was calculated to be 52.4 ± 0.8.

### 16S rRNA gene studies

**Fig. 2:**
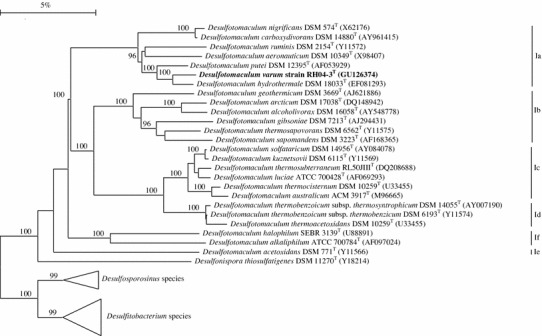
A dendrogram of the phylogenetic position of *Desulfotomaculum varum* strain RH04-3^T^ and its closest relatives. *Desulfotomaculum* clusters Ia to If. *Triangles* indicate outgroups and include: *Desulfosporosinus meridiei* DSM 13257^T^ (AF076527), *Desulfosporosinus auripigmenti* DSM 13351^T^ (AJ493051) and *Desulfosporosinusorientis* DSM 765^T^ (Y11570) representing members of the genus *Desulfosporosinus*, and *Desulfitobacterium metallireducens* DSM 15288^T^ (AF297871), *Desulfitobacterium dehalogenans* DSM 9161^T^ (L28946), *Desulfitobacteriumchlororespirans* DSM 11544^T^ (U68528) and *Desulfitobacterium hafniense* DSM 10664^T^ (U40078) representing members of the genus *Desulfitobacterium*. The *scale bar* indicates five nucleotide changes per 100 nucleotides, bootstrap values >95 are shown and GenBank accession numbers are given in *parentheses*

The phylogenetic analysis of the consensus 16S rRNA gene sequence comprising 1,479 nucleotides indicated that strain RH04-3^T^ was a member of the family *Peptococcaceae*, class *Clostridia*, phylum *Firmicutes* and was most closely related to *Desulfotomaculum putei* DSM 12395^T^ (similarity value of 95.2%) and *Desulfotomaculum hydrothermale* DSM 18033^T^ (similarity value of 93.6%) of genus *Desulfotomaculum* cluster Ia (Fig. 2). The defining signature nucleotides G:A at position 662/743, A:U at positions 1118/1115 and 1120/1153 (*E. coli* numbering according to Winkler and Woese 1991) and several other discriminatory nucleotides, distributed in a subcluster Ia at the exclusion of other members of the *Desulfotomaculum* subclusters, were also identified in the 16S rRNA gene sequence of strain RH04-3^T^ confirming its phylogenetic placement as a member of subcluster Ia (Table 3).

### Fatty acid composition

The whole-cell fatty acid profile of strain RH04-3^T^ revealed a proportionally high level of *iso*-C_15:0_, C_16:0_ and *iso*-C_17:0_, which is consistent with members of the *Desulfotomaculum* species (Ueki and Suto 1979; Hagenauer et al. 1997; Liu et al. 1997; Love et al. 1993), but quantitative differences in the fatty acid content were also observed in strain RH04-3^T^ compared to its nearest phylogenetic neighbors, *Desulfotomaculum hydrothermale* DSM 18033^T^ and *Desulfotomaculum putei* DSM 12395^T^ (Table 2). These differences include the presence of C_16:0 ALD_ and C_16:1 *c*11_ in strain RH04-3^T^ and *D. putei*, but not in *D. hydrothermale* (*Haouari* et al. 2008), and the presence of *iso*-C_16:0_, C_17:0_, and anteiso-C_17:0 DMA_ in strain RH04-3^T^ and *D. hydrothermale*, but not in *D. putei* (data also supported by *Haouari* et al. 2008). In addition, C_12:0_, C_15:0_, C_14:0 DMA_, C_17:0 DMA_, C_18:1 *c*13_, C_18:1 *c*9 DMA_, C_18:1 *c*11 DMA_, *iso*-C_11:0_, *iso*-C_13:0_, *iso*-C_14:0_, *iso*-C_15:0 DMA_ and *iso*-C_15:0 3OH_ were present in small quantities in strain RH04-3^T^, but not in *D*. *putei* and *D. hydrothermale.*

**Table 2:** Cellular fatty acid composition (%) of strain RH04-3^T^, *D. hydrothermale* DSM 18033^T^ and *D. putei* DSM 12395^T^

Fatty acid	1	2^a^	3^a^
Saturated straight chain			
12:0	0.3	–	–
14:0	3.9	3.0	0.9
15:0	0.4	–	–
16:0	19.8	17.7	10.7
17:0	0.5	–	1.0
18:0	2.4	1.2	1.9
Unsaturated straight chain			
14:0 DMA	0.2	–	–
15:0 DMA	–	0.6	–
16:1 *c*7	3.6	2.4	1.3
16:1 *c*9	8.5	8.7	3.6
16:1 *c*11	1.0	0.6	–
16:1 *c*9 DMA	1.3	2.0	0.9
16:0 DMA	3.9	7.1	3.5
17:0 DMA	0.1	–	–
17:0 cyclo	2.1	5.9	5.8
18:1 *c*9	1.0	0.7	0.8
18:1 *c*11	–	1.7	2.1
18:1 *c*13	0.5	–	–
18:1 *c*9 DMA	0.3	–	–
18:1 *c*11 DMA	0.4	–	–
Aldehyde			
16:0 ALD	0.7	1.1	–
Saturated branched chain			
*iso*-11:0	0.1	–	–
*iso*-13:0	0.3	–	–
*iso*-14:0	0.1	–	–
*iso*-15:0	19.0	20.5	15.7
*iso*-15:0 DMA	0.3	–	–
anteiso-15:0	3.3	1.2	2.0
*iso*-15:0 3OH	0.3	–	–
*iso*-16:0	0.6	–	0.9
*iso*-17:0	15.2	16.9	35.5
*iso*-17:0 DMA	2.7	8.2	8.5
anteiso-17:0	2.9	0.5	4.3
anteiso-17:0 DMA	0.5	–	0.6
Summed features			
4	0.2	–	–
6	0.2	–	–
8	0.3	–	–
10	3.2	–	–
Total (%)	100	100	100

All strains were grown under the same conditions

Strains: *1* strain RH04-3^T^, *2 Desulfotomaculum putei* DSM 12395^T^, *3Desulfotomaculum hydrothermale* DSM 18033^T^, *ALD* aldehyde, *DMA* dimethyl acetal, − negative

^a^Data obtained from Haouari et al. (2008)

## Discussion

Strain RH04-3^T^ exhibits phenotypic traits that are consistent with members of the genus *Desulfotomaculum*, which includes the Gram-positive nature of the cell wall ultrastructure, strictly anaerobic growth, the ability to form spores, sulfate reduction, a DNA G + C mol% content between 50 and 60% and the dominant presence of *iso*-C_15:0_, C_16:0_, C_18:0_ and *iso*-C_17:0_ in the whole-cell fatty acids. Strain RH04-3^T^ shares exclusively with its phylogenetic relatives in *Desulfotomaculum* cluster Ia the ability to form oval-shaped spores, growth in the absence of NaCl, fermentation of pyruvate, reduction of thiosulfate and utilization of H_2_ and lactate, but not malate, and the defining signature nucleotides G:A at position 662/743, A:U at position 1118/1115 and A:U at position 1120/1153 (*E. coli* numbering according to Winkler and Woese 1991) are consistent with the placement of strain RH04-3^T^ as a member of cluster Ia (Table 3). Strain RH04-3^T^ shares a few phenotypic characteristics with its nearest phylogenetic relatives, *D. hydrothermale* DSM 18033^T^ (*Haouari* et al. 2008) and *D. putei* DSM 12395^T^ (Liu et al. 1997), which include similar cell length and width, optimal growth at near neutral pH values, the ability to reduce sulfite but not nitrate and nitrite, and the inability to utilize glucose, lactose, acetate, propionate, benzoate, butyrate and succinate. However, the presence of a smaller helix 6 (positions 61–106, *E*. *coli* numbering according to Winkler and Woese 1991), comprising 32–34 nucleotides in strain RH04-3^T,^and *Desulfotomaculum putei* and a larger helix 6, comprising 80–100 nucleotides in *D. hydrothermale* (and the nine members of *Desulfotomaculum* clusters Ic and Id, and *D. geothermicum*, cluster Ib), clearly differentiate strain RH04-3^T^ from *D. hydrothermale*.

**Table 3:** 16S rDNA signature nucleotides in *Desulfotomaculum* subclusters Ia through to If

*E. coli* positions	*E. coli*	Signature nucleotide(s) in:					
		Subcluster Ia (7 strains)	Subcluster Ib (6 strains)	Subcluster Ic (6 strains)	Subcluster Id (3 strains)	Subcluster Ie (1 strain)	Subcluster If (2 strains)
66–103	A–U	A–U	A–U	G–C	G–C	A–U	A–U
154–167	U–A	A–U	G–U	G–C G–G	Pur–Pyr	G–U	A–U U–A
157–164	U–G	U–G	G–Pyr	G–U	G–Pyr	G–C	G–C
184	A	C	C	C	C	A	C
252–274	U–A	U–A	U–A	A–U	A–U	U–A	U–A
259–267	G–C	U–A	G–C	G–C	G–C	G–C	G–C U–A
293–304	G–U	G–C	G–C	Pur–U	G–U	G–U	G–Pyr
320–333	A–U	G–C	A–U	G–C	G–C	A–U	Pur–Pyr
381	C	A	C	A	A	A	A
409–433	U–G	G–C	Pyr–Pur	C–G	C–G	C–G	G–C
427	U	U	U	C	C	U	U A
440–494	C–G	Pyr–G	C–G	U–G	U–G	G–G	U–G [y–G]
444–490	G–C	A–U	G–C A–U A–A	G–C	G–C	U–A	U–A
449	G	A	A	A	C	C	A
534	U	A	A	G	G	U	C
580	C	C	U	C	C	A	C
582	C	U	U	U	C	U	U
586–755	C–G	U–A	C–G	C–G	C–G	U–A	C–G
589–650	U–G	U–A	C–G	Pyr–Pur	C–G	U–A	Pyr–Pur
593–646	U–G	U–G	G–Pyr	A–U	A–U	U–G	U–G
603–635	U–A	G–C	A–U T–G	G–C	G–C	A–T	G–C
610–630	U–A	G–A	C–C	U–G	U–G	A–A	G–Pur
657–749	U–A	U–A	U–A	G–C	G–C	U–A	U–A
662–743^a^	U–A	G–A	G–C A–C T–C	G–Pyr	G–C	G–C	G–C
672	U	C	U	U	U	U	U
681–709	A–U	C–G	C–G	C–G	C–G	U–A	Pyr–A
682–708	A–U	A–U	A–U	G–C	G–C	A–U	A–U
730	G	G	A	G	G	G	G
738	C	U	C	U	U	C	C
776	G	G	U	G	G	G	U
778	G	G	G	G	G	U	G
822–878	U–A	A–U	G–Pyr	G–C	G–C	A–U	A–U
896–903	C–G	U–A^b^	C–G	C–G	C–G	U–A	U–A
948–1233	C–G	U–A	G–A C–G	C–G	C–G	U–A	C–G
989–1216	U–A	G–U	U–A	G–U	G–U	G–U	Pur–U
1217	C	C	U	C	C	C	C
1118–1155^a^	U–A	A–U	C–G	C–G	C–G	C–G	C–G
1120–1153^a^	C–G	A–U	U–A	G–C	G–C	G–C	G–C
1129–1143	C–G	C–G	C–G	U–A	C–G	C–G	C–G
1210	C	C	U	C	U	C	C
1253–1284	G–C	C-G	C–G	G–C	G–C	G–C	G–C
1310–1327	G–C	U–A	C–U C–G	C–G	C–G	U–A	C–G

Subcluster Ia is composed of: *D. nigrificans* DSM 574^T^ (X62176), *D. carboxydivorans* DSM 14880^T^ (AY961415), *D. ruminis* DSM 2154^T^ (Y11572), *D. aeronauticum* DSM 10349^T^ (X98407), *D. putei* DSM 12395^T^ (AF053929), *D. varum* strain RH04-3^T^ (GU126374) and *D. hydrothermale* DSM 18033^T^ (EF081293). Subcluster Ib is composed of: *D. geothermicum* DSM 3669^T^ (AJ621886), *D. arcticum* DSM 17038^T^ (DQ148942), *D. alcoholivorax* DSM 16058^T^ (AY548778), *D. gibsoniae* DSM 7213^T^ (AJ294431), *D. thermosapovorans* DSM 6562^T^ (Y11575) and *D. sapomandens* DSM 3223^T^ (AF168365). Subcluster Ic is composed of: *D.solfataricum* DSM 14956^T^ (AY084078), *D. kuznetsovii* DSM 6115^T^ (Y11569), *D.thermosubterraneum* RL50JIII^T^ (DQ208688), *D. luciae* ATCC 700428^T^ (AF069293), *D. thermocisternum* DSM 10259^T^ and *D. australicum* ACM 3917^T^ (M96665). Subcluster Id is composed of: *D. thermobenzoicum* subsp. *thermosyntrophicum* DSM 14055^T^ (AY007190), *D. thermobenzoicum* subsp. *thermobenzicum* DSM 6193^T^ (Y11574) and *D. thermoacetoxidans* DSM 10259^T^ (U33455). Subcluster Ie is composed of *D. acetoxidans* DSM 771^T^ (Y11566). Subcluster If is composed of *D. halophilum* SEBR 3139^T^ (U88891) and *D. alkaliphilum* ATCC 700784^T^ (AF097024)

*Pur* purine, *Pyr* Pyrimidine

^a^Subcluster Ia-specific nucleotide positions

^b^*E. coli* position 903 for *D. nigrificans* DSM 574^T^ (X62176) is not defined (N)

Strain RH04-3^T^ also has many traits that clearly differentiates it from *D. hydrothermale* DSM 18033^T^ and *D. putei* DSM 12395^T^, the two nearest phylogenetic neighbors. The similarity of strain RH04-3^T^ to *D. hydrothermale* DSM 18033^T^ and *D. putei* DSM 12395^T^ is only 93.6 and 95.2% respectively, the temperature optima for growth, the temperature growth range, pH growth range, yeast extract requirement for growth, mol % G + C content of the DNA, range of electron acceptors used, the range of substrates used (Table 4), and the whole-cell fatty acid composition (Table 2) also differentiate the three isolates. Based on the phenotypic and phylogenetic differences, we suggest that strain RH04-3^T^ represents a novel species for which the name *Desulfotomaculum varum* sp. nov., is proposed.

**Table 4:** Differential characteristics of strain RH04-3^T^ and its closest relatives

Characteristic	1	2	3
Geographical location	Microbial mats from a thermal aquifer bore well outflow	Siltstone from a deep terrestrial subsurface	Hot spring
Habitat	Great Artesian Basin, Australia	Tayolrsville Triassic Basin, USA	Hamam Essalhine hot spring, Tunisia
Morphology	Straight to slightly curved rod-shaped	ND	Slightly curved rod shaped
Length (μm)	2.0–5.0	2.0–5.0	3.0–6.0
Width (μm)	1.0	1.0–1.1	1.0
Gram-stain	Positive	Negative	Negative
Cell-wall ultrastructure	Negative	Positive	Positive
Temperature growth range ( °C)			
Optimum	50	64	55
Range	37–55	40–65	40–60
pH growth range			
Optimum	7	7.5	7
Range	5–8.5	6–7.9	5.8–8.2
NaCl growth range [% (w/v)]	0–1	0–2	0–1.5
Yeast extract requirement	+	ND	**–**
Utilization of electron donors with sulfate			
Fructose	+	+	**–**
Mannose	+	ND	**–**
Ethanol	**–**	+	+
Butanol	**–**	ND	+
Methanol	**–**	+	**–**
Propanol	**–**	ND	+
Formate	**–**	+	+
Pyruvate	+	+	+
Substrates fermented			
Fructose	**–**	+	**–**
Electron acceptors			
Iron(III)	**–**	**–**	+
As(V)	**–**	ND	+
DNA G + C content (mol %)	52.4^a^	47.1^b^	46.9^b^
16S rRNA similarity to RH04-3^T^ (%)	100	95.2	93.6

Strains: *1* strain RH04-3^T^, *2 Desulfotomaculum putei* (Liu et al. 1997), *3Desulfotomaculum hydrothermale* (*Haouari* et al. 2008), + positive, − negative, *ND* not determined

^a^Determined by the thermal denaturation method

^b^Determined by HPLC

### Description of *Desulfotomaculum varum* sp. nov

*Desulfotomaculm varum* (*va’. rum* M. L. adj varum bent, bow legged pertaining to the slightly curved cell shape).

Cells are strictly anaerobic, thermophilic, straight to slightly curved rods (2.0–5.0 × 1.0 μm) that stain Gram-positive and possess a Gram-positive cell wall ultrastructure. Cells possess peritrichous flagella and produce oval, subterminal to terminal spores. Growth occurs between 37 and 55 °C (optimum temperature 50 °C) and pH 5.0–8.5 (optimum pH 7). Sulfate, sulfite, thiosulfate and elemental sulfur are used as electron acceptors but not fumarate, nitrate, nitrite, iron(III), arsenate(V), vanadium(V) or cobalt(III). Electron donors used in the presence of sulfate as an electron acceptor include glycerol, fructose, mannose, lactate, pyruvate and H_2_, but not glucose, lactose, ethanol, malate, acetate, propionate, formate, benzoate, succinate, butyrate, casamino acids, propanol, methanol and butanol. In the absence of sulfate, pyruvate was fermented, but not fructose, mannose, glycerol, lactate, acetate, malate, formate, ethanol, butanol and propanol. Sensitive to chloramphenicol, streptomycin, tetracycline, penicillin, ampicillin and NaCl concentrations greater than 1.5%. The G + C content of the genomic DNA is 52.4 ± 0.8 mol %. Phylogenetically related to members of the genus *Desulfotomaculum*, most closely to *Desulfotomaculum hydrothermale* (16S rRNA similarity of 93.6%) and *Desulfotomaculum putei* (16S rRNA similarity of 95.2%). The type strain RH04-3^T^ (= KCTC = 5794^T^ = JCM 16158^T^) was isolated from a microbial mat sample from the outflow of a Great Artesian Basin bore well (the New Lorne Bore) in Queensland, Australia.
